# Fascin-1 expression is associated with neuroendocrine prostate cancer and directly suppressed by androgen receptor

**DOI:** 10.1038/s41416-023-02449-x

**Published:** 2023-10-24

**Authors:** Anthony Turpin, Carine Delliaux, Pauline Parent, Hortense Chevalier, Carmen Escudero-Iriarte, Franck Bonardi, Nathalie Vanpouille, Anne Flourens, Jessica Querol, Aurélien Carnot, Xavier Leroy, Nicolás Herranz, Tristan Lanel, Arnauld Villers, Jonathan Olivier, Hélène Touzet, Yvan de Launoit, Tian V. Tian, Martine Duterque-Coquillaud

**Affiliations:** 1 University Lille, CNRS, INSERM, CHU Lille, Institut Pasteur de Lille, UMR9020-U1277-CANTHER—Cancer Heterogeneity Plasticity and Resistance to Therapies, F-59000 Lille, France; 2 Department of Medical Oncology, Lille University Hospital, F-59000 Lille, France; 3 Department of Medical Oncology, Centre Oscar Lambret, 3, rue Frederic Combemale, 59000 Lille, France; 4 Vall d’Hebron Institute of Oncology (VHIO), 08035 Barcelona, Spain; 5 University Lille, CNRS, Inserm, CHU Lille, Institut Pasteur de Lille, US 41 - UAR 2014 - PLBS, F-59000 Lille, France; 6 Institut de Pathologie, CHU Lille, Avenue Oscar Lambret, F-59000 Lille, France; 7 Department of Urology, Hospital Claude Huriez, CHU Lille, Lille, France; 8 University Lille, CNRS, Centrale Lille, UMR 9189 CRIStAL, F-59000 Lille, France

**Keywords:** Cancer, Endocrine cancer

## Abstract

**Background:**

Neuroendocrine prostate cancer (NEPC) is an aggressive form of prostate cancer, arising from resistance to androgen-deprivation therapies. However, the molecular mechanisms associated with NEPC development and invasiveness are still poorly understood. Here we investigated the expression and functional significance of Fascin-1 (FSCN1), a pro-metastasis actin-bundling protein associated with poor prognosis of several cancers, in neuroendocrine differentiation of prostate cancer.

**Methods:**

Differential expression analyses using Genome Expression Omnibus (GEO) database, clinical samples and cell lines were performed. Androgen or antagonist’s cellular treatments and knockdown experiments were used to detect changes in cell morphology, molecular markers, migration properties and in vivo tumour growth. Chromatin immunoprecipitation-sequencing (ChIP-Seq) data and ChIP assays were analysed to decipher androgen receptor (AR) binding.

**Results:**

We demonstrated that *FSCN1* is upregulated during neuroendocrine differentiation of prostate cancer in vitro, leading to phenotypic changes and NEPC marker expression. In human prostate cancer samples, FSCN1 expression is restricted to NEPC tumours. We showed that the androgen-activated AR downregulates *FSCN1* expression and works as a transcriptional repressor to directly suppress *FSCN1* expression. AR antagonists alleviate this repression. In addition, FSCN1 silencing further impairs in vivo tumour growth.

**Conclusion:**

Collectively, our findings identify *FSCN1* as an AR-repressed gene. Particularly, it is involved in NEPC aggressiveness. Our results provide the rationale for the future clinical development of FSCN1 inhibitors in NEPC patients.

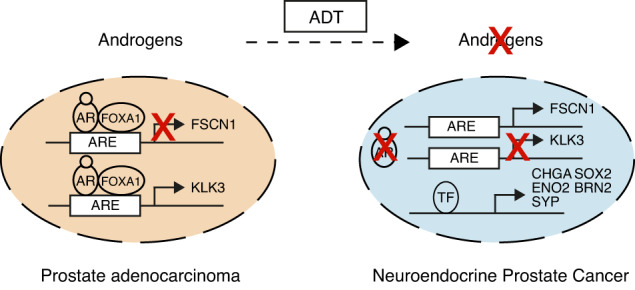

## Background

Prostate cancer (PCa) is the most common cancer in males worldwide. Early detection of the disease and local therapies, including surgical and radiation treatments, have improved the overall survival of PCa patients [1]. However, most PCa mortalities are caused by the metastatic progression of the disease [2]. Because PCa is driven by androgen through the nuclear androgen receptor (AR), androgen-deprivation therapy (ADT) alone or combined with radiotherapy or chemotherapy has been used as routine treatment. However, despite the initial high response rate to ADT, nearly all patients evolve to castrate-resistant prostate cancer (CRPC) associated with poor prognosis [3, 4]. Patients with CRPC develop resistance arising from multiple molecular mechanisms, such as increased androgen biosynthesis in the tumour microenvironment or through alterations of AR signalling, including AR mutations, constitutively active AR variants in the absence of ligands, AR gene amplifications, use of other signalling pathways or reliance on non-AR-mediated pathways [3, 5, 6]. Moreover, it has been shown that a small proportion of CRPC tumour cells acquire a neuroendocrine phenotype with neuronal marker expression, including chromogranin A (CHGA), synaptophysin (SYP) or enolase-2 (ENO2) [7, 8].

Neuroendocrine prostate cancer (NEPC), rarely arising de novo, is a highly aggressive form of PCa with a proliferative area of the tumour mass and metastasis progression [9, 10]. The origin of NEPC has been suggested to arise from the selection pressure exerted by ADT, inducing the neuroendocrine transdifferentiation of CRPC cells [11–13]. A hallmark of NEPC is the loss of AR signalling during neuroendocrine transdifferentiation, resulting in resistance to ADT [14]. Therefore, with the introduction of AR-targeted therapies, such as enzalutamide, a second-generation non-steroidal AR inhibitor [15], the incidence of NEPC is expected to increase [16, 17].

Interestingly, in our previous studies, we identified a series of genes involved in the metastasis progression [18–20]. Among the identified genes, we selected the *Fascin-1* (*FSCN1*) gene originally identified as a gene that encodes an actin-bundling protein known to stabilise filopodia and invadopodia by regulating the parallel bundling of actin filaments [21, 22]. FSCN1 is a key regulator of cell cytoskeleton remodelling, which drives cell adhesion, migration and invasion [23]. FSCN1 is not expressed in most adult human epithelia, but high FSCN1 expression has been associated with increased mortality in breast, ovarian, colorectal and pancreatic carcinomas and metastasis [24–27].

In PCa, FSCN1 expression has been reported in PCa stromal cells in tumours with high Gleason scores [28] and in tumour cells in a CRPC sample [29]. Given these intriguing reports, we, therefore, assessed *FSCN1* involvement in PCa progression. In this study, we report that the *FSCN1* gene is repressed by AR in androgen-dependent cell lines. Moreover, *FSCN1* expression, absent in primary PCa, is detected in NEPC tumours and may be involved in tumour progression. Our study identifies FSCN1 as a new marker of NEPC and a potential mediator of invasiveness.

## Methods

### Cell lines and treatments

Human PCa cell lines VCaP, LNCaP, MDA-PCA-2b, DU145, NCI-H660 and PC3 were obtained from the American Type Culture Collection (ATCC, Manassas, VA, USA). Cell lines were grown in ATCC-recommended media supplemented with 10% foetal bovine serum (FBS, Thermo Fisher Scientific, Waltham, MA, USA). PC3M-luc-C6 (PC3M) cell line was purchased from Caliper (Perkin Elmer, Hopkinton, MA, USA). The PC3c cell line was obtained from Edith Bonnelye and cultured as described in ref. [30]. PC3c clones, stably transfected with either an empty pcDNA3.1 vector or a *TMPRSS2:ERG* (T1E4) expression vector, were obtained as described previously [18]. All cells were subcultured every 3–4 days and maintained in a humidified incubator at 37 °C and 5% CO_2_. Cells were not used beyond 15 passages; they were tested for mycoplasma contamination with the MycoAlert™ mycoplasma detection kit every 6 months (Lonza, Basel, Switzerland). To activate the androgen receptor, VCaP and LNCaP cells were respectively treated with 100 nM and 10 nM of dihydrotestosterone (DHT, Sigma-Aldrich, St. Louis, MO, USA) for 2 or 16 h after being hormone-starved using charcoal-depleted serum containing the medium for 8 h. The activity of AR was inhibited with 50 µM of bicalutamide (BIC) (Sigma-Aldrich).

### Transfection of constructs or siRNA in PC3, LNCaP and VCaP cells

Predesigned and pooled small interfering RNAs (siRNAs) (siFSCN1: ON-TARGETplus human FSCN1 siRNA) and the control siRNA (ON-TARGETplus Non-targeting Pool D001810-10) were obtained from GE Dharmacon (Lafayette, CO, USA). Cells were transfected with siRNA (50 nM) using the Lipofectamine 2000 reagent (Thermo Fisher Scientific) according to the manufacturer’s instructions. Gene-knockdown or overexpression effects were evaluated 72 h after transfection. Transient overexpression experiments involved cell transfection with the AR expression plasmid (PSG5-AR) (4 µg) or with the empty vector (PSG5) using the Lipofectamine 2000 reagent.

#### Generating stable *FSCN1* knockdown cell lines

PC3M prostate cells were maintained using modified Eagle’s medium (MEM) (Biowest, L0440-500) supplemented with 1 mM sodium pyruvate (Fisher scientific, 11530396), 1% penicillin–streptomycin (Gibco; 15140122) and 10% foetal bovine serum (FBS) (Gibco; 10270106) at 37 °C in 5% CO_2_. PC3M cells were transduced with lentivirus containing either an irrelevant short hairpin (shScramble) (Sigma-Aldrich SHC002V) or two different shFSCN1 (Sigma-Aldrich, TCR000012342, and TRCN0000123039) and selected with puromycin 1 μg/mL (Fisher Bioreagents, BP2956-100).

For lentiviral production, HEK293T cells were transfected by adding a mixture of 150 mM NaCl, DNA (50% of the indicated shRNA vector, 10% pCMV-VSVG, 30% pMDLg/pRRE, and 10% pRSV rev) and polyethyleneimine polymer (Polysciences Inc; 23966-1), which was previously incubated 15 min at room temperature. After 24 h, the transfection medium was removed and changed to a fresh medium in which viruses were produced. The virus-containing medium was then collected and filtered with a 0.45-μm filter unit (Merck Millipore; 051338) at 24 and 48 h. The virus-containing medium was used to infect PC3M cells, as mentioned above.

#### Cell growth and migration assays

For MTT (3-(4,5-dimethylthiazol-2-yl)-2,5-diphenyltetrazolium bromide) assay, 1000 PC3M cells expressing shScramble, shFSCN1.2, or shFSCN1.3 were seeded in a well of a 96-well plate, and cell growth for 7 days. Cells were incubated with 0.3 mg of MTT (Panreac Aplichem, A2231) per mL of culture medium without FBS for 3 h at 37 °C. Subsequently, the MTT-containing medium was removed, and cells were incubated with isopropanol. The absorbance was measured at 565 nm with infinite M2000 Pro (Tecan), and data were acquired using Tecan i-control 1.11 software.

For migration assay, 20,000 cells of PC3M shScramble, shFSCN1.2 or shFSCN1.3 were seeded in MEM without FBS in transwell filter chambers (Corning, 3422) in triplicate. One hour after the seeding, MEM with 10% FBS was added to the lower chamber as a chemoattractant, and cells were kept at 37 °C for 24 h. After removing non-migrating cells from the upper surface of the membrane, migrating cells were fixed with paraformaldehyde 4% for 15 min, and nuclei were stained with 0.25 μg/mL of PBS-DAPI (4′,6-diamidino-2-phenylindole). Finally, nuclei were counted using an epifluorescence microscope and ImageJ software.

#### Tumour xenografts

To assess tumour growth capacity, 3 × 10^6^ cells of either PC3M shScramble, shFSCN1.2 or shFSCN1.3 were subcutaneously implanted per flank in NOD-SCID mice. Tumour growth and mice weight were measured twice per week. Tumour volumes were calculated using the formula: V = (length × width^2^)/2. According to institutional guidelines, mice were euthanized using CO_2_ inhalation once the experiment reached the endpoint, and tumours were surgically extracted and individually weighted. All aminal procedures were approved by the Ethics Committee of Animal Experimentation (CEEA) at the Vall d’Hebron Institute of Research and by the Catalan Government.

### RNA extraction and gene expression analysis

Total RNA was extracted from cells using the NucleoSpin RNA Plus kit (Macherey-Nagel, Düren, Germany) according to the manufacturer’s instructions. For retrotranscription, 1 µg of total RNA was used to generate cDNA using the High-Capacity RNA-to-cDNA kit (Applied Biosystems, Foster City, CA, USA). Real-time quantitative PCRs (qPCRs) were performed using the Power SYBR^TM^ Green PCR Master kit (Applied Biosystems, Foster City, CA, USA) on a Stratagene Mx3005P qPCR System (Stratagene, La Jolla, CA, USA) according to the manufacturer’s instructions. The relative expression levels of individual genes were calculated using the -2ΔΔCT method. All reactions were normalised to human *GAPDH* or *L32* genes and ran in triplicate. Optimal primer specificity and efficiency were validated according to the Mx3005P qPCR system user’s guide. The primers used in this study can be found in Supplementary Table S1.

### Western blotting and ChIP

Cell proteins were extracted and separated in 4–10% SDS-PAGE. After migration, samples were transferred by electrophoresis to PVDF membranes (Transfert-blot Turbo Transfer system, Bio-Rad, Hercules, CA, USA). Membranes were incubated with blocking buffer for 3 h, then with the primary antibody overnight. Membranes were incubated with secondary HRP-conjugated antibodies for 1 h. After washing, membranes were revealed using Clarity™ Western ECL substrate (Bio-Rad). Antibodies used were as follows: anti-FSCN1 (ab126772, Abcam Epitomics, Cambridge, UK), anti-AR (sc-816, N-20 Santa Cruz Biotech, Dallas, TX, USA), anti-vinculin (Invitrogen VIN-01, Thermo Fisher Scientific), anti-GAPDH (sc-32233 Santa Cruz Biotech or (ab128915, Abcam) and anti-β-actin (Clone AC-15, Sigma-Aldrich).

Chromatin immunoprecipitation (ChIP) assays were performed as described [20] using either a rabbit polyclonal anti-AR antibody (AR sc-7305, Santa Cruz Biotech), a rabbit polyclonal anti-FOXA1 ChIP-grade antibody (ab23738, Abcam) or a control rabbit IgG antibody (Rabbit DA1E Ab IgG XP®, Cell Signaling Technology, Danvers, MA, USA). The genomic DNA was purified using NucleoSpin Clean-up columns (Macherey-Nagel) according to the manufacturer’s instructions. Immunoprecipitated genomic DNA was analysed using qPCR, and the primers used in this study are given in Supplementary Table S2.

### Human prostate cancer samples

Human PCa samples (*n* = 52) were obtained from the local tumour tissue bank (Tumorothèque Alliance Cancer, Lille, France) after approval by the internal review board (CSTMT-042, 27/07/2009). As described by Tian et al. [18], these tumour tissue samples, which had been extracted and subsequently frozen, originated from radical prostatectomies or transurethral prostatic resections performed at Lille University Hospitals (CHU de Lille). In addition, four NEPC tissues were collected and sectioned to analyse the expression of *FSCN1* (Supplementary Table S3). All patients were informed, and consent was obtained by the referring physician.

### Bioinformatic analysis

To analyse *FSCN1* and *KLK3* mRNA expression levels in normal and adjacent prostate tissues or primary and metastatic prostate cancer samples, expression microarray data were downloaded from the GEO database with accession numbers GSE35988, GSE6919, GSE8511, GSE3325 [31, 32].

To determine the recruitment of AR on the promoters of *FSCN1* and *KLK3*, we analysed publicly available ChIP-seq data (GSE55062, GSE43791, GSE40050, GSE62442, GSE61838, GSE32356, GSE56288) for AR binding in untreated, DHT-, BIC- or ENZ-treated PCa cells and in patient prostate normal and tumour samples. AR binding was then visualised on the UCSC browser (http://www.genome.ucsc.edu) [33]. Tracks from the human ENCODE project for H3K27ac and H3K4me1 marks, often found near active enhancer elements, and H3K4me3 effects, often found near active promoters well as DNase I hypersensitivity peak clusters, have been added. The same process was done to determine the recruitment of FOXA1, along with AR, on the promoters of *FSCN1* and *KLK3*, using publicly available ChIP-seq data (GSE94682, GSE83860, GSE58428, GSE56046, GSE161948).

To visualise the enhancer-associated histone modification H3K27-acetylated (H3K27ac) at the *FSCN1*, *KLK3* and *CHGA* genes, H3K27ac profiles of five representative prostate cancer adenocarcinoma (PRAD) and five neuroendocrine prostate cancer (NEPC) patient-derived xenografts (PDXs) were extracted from ref. [34].

### Immunohistochemistry and immunofluorescence

Immunohistochemistry of human tissue sections was performed using anti-FSCN1 at 1:100 (ab49815 Abcam and FCN01 55K-2 Thermo Fisher). Sections were incubated with secondary HRP-conjugated antibodies. Counterstaining was performed using Mayer’s hematoxylin (Merck, Darmstadt, Germany).

Immunofluorescence assays were performed as described by Tian et al. [18]. The antibodies used in this study were anti-FSCN1 at 1:100 and Alexa Fluor^TM^ 488-conjugated anti-rabbit IgG (Invitrogen, dilution 1:500). Images were acquired using an LSM 710 confocal microscopy system and ZEN 2010 software (Carl Zeiss, Oberkochen, Germany).

### Fluorescent gelatine degradation assay

Poly-D-lysine-coated coverslips were washed with PBS and fixed with 0.5% glutaraldehyde for 15 min. Then, the coverslips were inverted on a 100-μl drop of gelatin conjugated with FITC (fluorescein, G13187 Invitrogen, Thermo Fisher Scientific) and incubated for 10 min in the dark. After washing with PBS, the residual reactive groups were quenched with 5 mg/ml sodium borohydride for 10 min and washed with PBS. Cells were plated in 24-well plates containing a coverslip coated with a fluorescent gelatin matrix and incubated at 37 °C. After 3 h, cells were fixed with 4% formaldehyde, permeabilised with 1% BSA with 10% normal goat serum, 0.3 mM glycine and 0.1% TBS-Tween. Then, cells were labelled for filamentous actin with Alexa Fluor^TM^ 555 phalloidin (8953 S, Cell Signaling Technology) and Hoechst (H33258, Sigma-Aldrich). Confocal images were acquired using the Olympus FV500 confocal laser scanning microscope and FluoView software (Olympus). Sites of matrix degradation were visible as dark areas (spots) in the bright green fluorescent gelatin matrix.

### Statistical analysis and image processing

Statistical analyses were performed using the GraphPad Prism software (San Diego, CA, USA). The statistical methods used in this study are indicated in the corresponding figure legends. All image processing was carried out using ImageJ.

## Results

### *FSCN1* expression in PCa cells is suppressed by androgen receptor

Using an AR-independent cell line (PC3c derived from the PC3 cell line), in a previous study which aimed to define the role of the ERG fusion (T1E4), the most frequent chromosomal rearrangement in PCa (> 50%), we identified a series of genes potentially involved in cell migration and invasion [18]. Among these genes, we showed that *FSCN1* is highly expressed in PC3c clones, which stably expresses the ERG-T1E4 fusion. This association suggested that *FSCN1* expression was regulated by the fusion (Supplementary Fig. S1a). However, when we examined *FSCN1* expression in a series of human primary prostate cancer (*n* = 52), fusion-positive (*n* = 32) or fusion-negative (*n* = 20) and normal prostate tissues (*n* = 20), we observed that no significant differences between these groups of samples (Supplementary Fig. S1b) and no correlation with the ERG transcription factor status. Therefore, ERG fusion is not the key regulator of *FSCN1* in PCa.

**Fig. 1:**
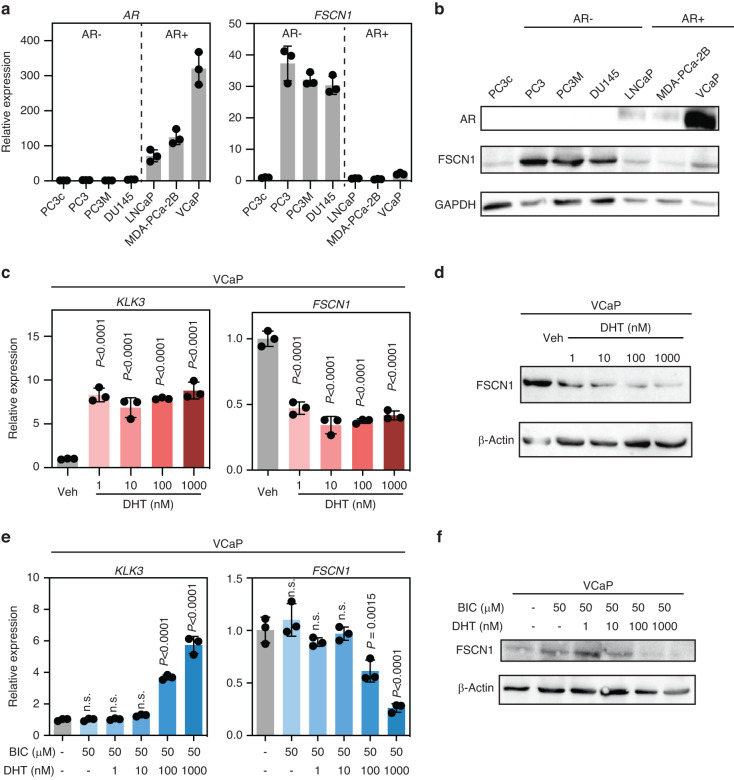
The *FSCN1* gene is downregulated by the androgen receptor in VCaP cells. **a** RT-PCR evaluating expression levels of *FSCN1* (right panel) from multiple cell lines (PC3c, PC3, PC3M-Luc, DU145, LNCaP, MDA-PCa-2b, VCaP) in which androgen receptor (*AR*) expression (left panel) was measured in parallel. Data were normalised to *FSCN1* and *AR* levels in PC3c cells. **b** Western blot validating expression of AR and FSCN1 proteins in cell lines. GAPDH was used as a loading control. **c** RT-PCR showing *FSCN1* expression in VCaP cells (right panel) according to AR activation in the presence of increasing doses (1–1000 nM) of dihydrotestosterone (DHT). Prostate-specific antigen (*KLK3)* expression (left panel) was a positive control of AR activation in the same experiment. Error bars indicate *n* = 3, mean +/− s.d. Statistical significance was determined by one-way ANOVA with a Dunnett multiple comparison test. **d** Western blot validating expression of FSCN1 in VCaP cells. β-actin was used as a loading control. **e** RT-PCR showing *FSCN1* expression in VCaP cells (right panel) according to AR activation or repression respectively in the presence of increasing doses (1–1000 nM) of dihydrotestosterone (DHT) administered in the presence or the absence of 50 µM bicalutamide (BIC). Prostate-specific antigen (*KLK3)* expression (left panel) was a positive control of AR activation or repression in the same experiment. Error bars indicate mean +/− s.d. Statistical significance was determined by one-way ANOVA with a Dunnett multiple comparison test (*n* = 3). **f** Western blot validating expression of *FSCN1* in VCaP cells. β-actin was used as a loading control. RT-PCR experiments were normalised to human *L32* genes and run in triplicate.

We then explored *FSCN1* expression in various PCa cell lines (Fig. 1a). Interestingly, except in the PC3c cell line, which differs from PC3 in its strong osteomimicry properties, *FSCN1* turned out to be highly expressed in cell lines that are negative for AR expression (Fig. 1a, b), such as PC3, PC3M and DU145, but not in any of the androgen-dependent cell lines such as LNCaP, MDA-PCa-2b and VCaP, suggesting that AR plays a role in the regulation of *FSCN1* expression in PCa cells. To confirm the androgen inhibition effect of *FSCN1* expression, we performed RT-qPCR and Western blot analyses of AR-positive VCaP cells treated with either 1–1000 nM of the AR activator DHT alone (Fig. 1c), or in combination with 50 μM of BIC, an AR antagonist (Fig. 1e). We used *KLK3* gene encoding PSA (prostate-specific antigen), known to be an AR-activated target gene, as a positive control, and successfully showed that DHT treatment is associated with a decrease in *FSCN1* expression at the mRNA level. Using WB analyses, we demonstrated that the expression of FSCN1 decreased upon DHT treatment (Fig. 1d, f), and this inhibition was partially recovered by treatment with BIC. Our data showed that androgens inhibit *FSCN1* expression through AR signalling.

### *FSCN1* is upregulated in NEPC

**Fig. 2:**
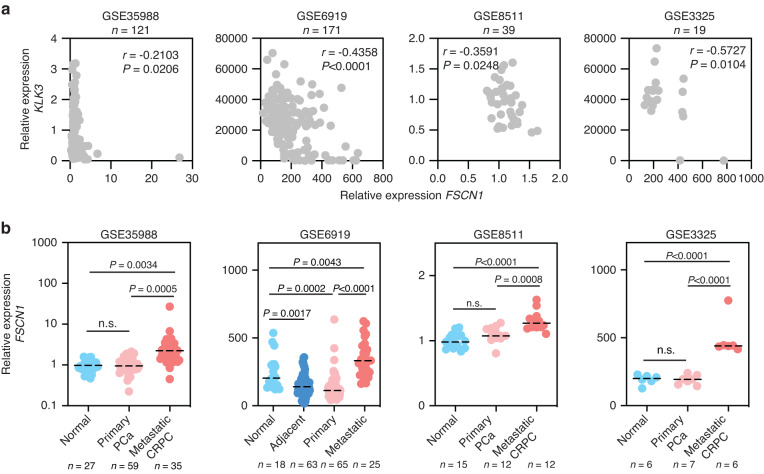
Expression of *FSCN1* in normal and cancerous human prostate. **a** Scatter plots showing correlation of relative gene expression between *FSCN1* and *KLK3* in gene expression datasets GSE35988, GSE6919, GSE8511 and GSE3325. Pearson’s test was used to determine the correlation coefficient and statistical significance. **b** Comparison of *FSCN1* expression profiles in normal prostate (normal or adjacent), primary prostate cancer (primary PCa) and in metastatic prostate cancer (metastatic PCa or CRPC) cells using gene expression datasets GSE35988, GSE6919, GSE8511, GSE3325. Statistical significance was determined by one-way analysis of variance (ANOVA) with a Tukey multiple comparison test.

To gain insight into the potential role of *FSCN1* in PCa, we investigated the *FSCN1* expression in normal, primary and metastatic PCa cohorts using published datasets (Fig. 2). We analysed *FSCN1* expression in published transcriptomic datasets from four independent PCa cohorts [35–38]. First, we observed an inverse correlation between *FSCN1* and *KLK3* expression, suggesting that a decrease of AR activity, corresponding to a low *KLK3* expression, is associated with an increased expression of *FSCN1* (Fig. 2a). Second, as shown in Fig. 2b, compared with normal or primary PCa samples, *FSCN1* expression levels were significantly higher in metastatic PCa samples [39–46]. This difference in expression suggests its potential role in metastasis.

**Fig. 3:**
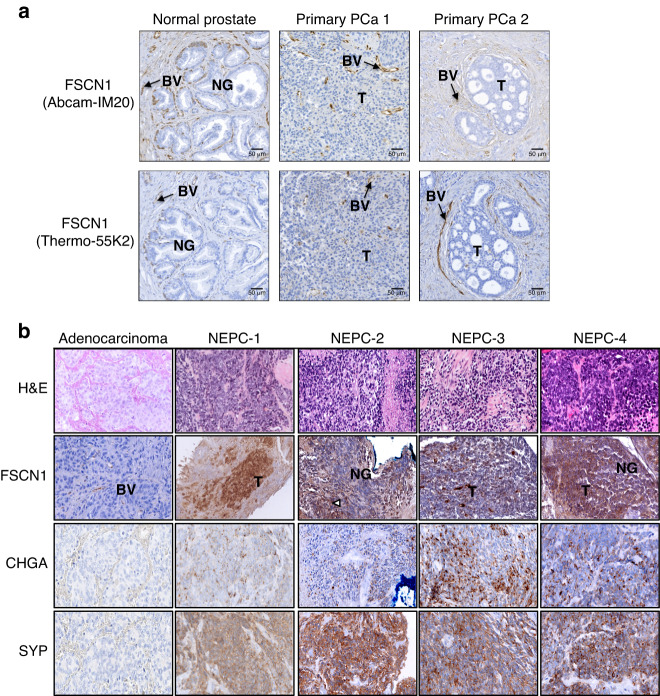
FSCN1 is highly expressed in human NEPC cells. **a** IHC staining was performed on normal prostate and primary PCa (PCa 1 and PCa 2), using two distinct antibodies against FSCN1 (Abcam-IM20 and Thermo-55K1). BV blood vessels, NG normal glands, T tumour. Scale bar = 50 µm. **b** Representative images showing hematoxylin–eosin-saffron (HES) and immunohistochemical staining for FSCN1, CHGA and SYP proteins in adenocarcinoma and four NEPC tumour samples (NEPC-1 to −4).

To further test the in vivo validity of the gene expression correlations from the PCa datasets, immunohistochemical assays of FSCN1, using two independent antibodies (Fig. 3a and Supplementary Fig. S2), were used and showed that its expression was only detectable in blood vessels, either in primary prostate tumours or in lymph node and bone metastasis, samples were already described in [18, 20]. Therefore, in accordance with our in vitro study in which we observed that AR-negative cell lines express *FSCN1* and the analysis of the PCa datasets, we hypothesised that *FSCN1* expression is specifically associated with NEPC. In Fig. 3b and Supplementary Table S3, using NEPC samples that were positive for neuroendocrine markers, such as CHGA and SYP, we detected the FSCN1 protein in NEPC cancer cells. By contrast, FSCN1 was undetectable in any adenocarcinoma studied, as exemplified in Fig. 3a, b. Taken together, these results suggest that FSCN1 is enriched in NEPC.

### Androgen deprivation triggers *FSCN1* expression in androgen-sensitive cells

**Fig. 4:**
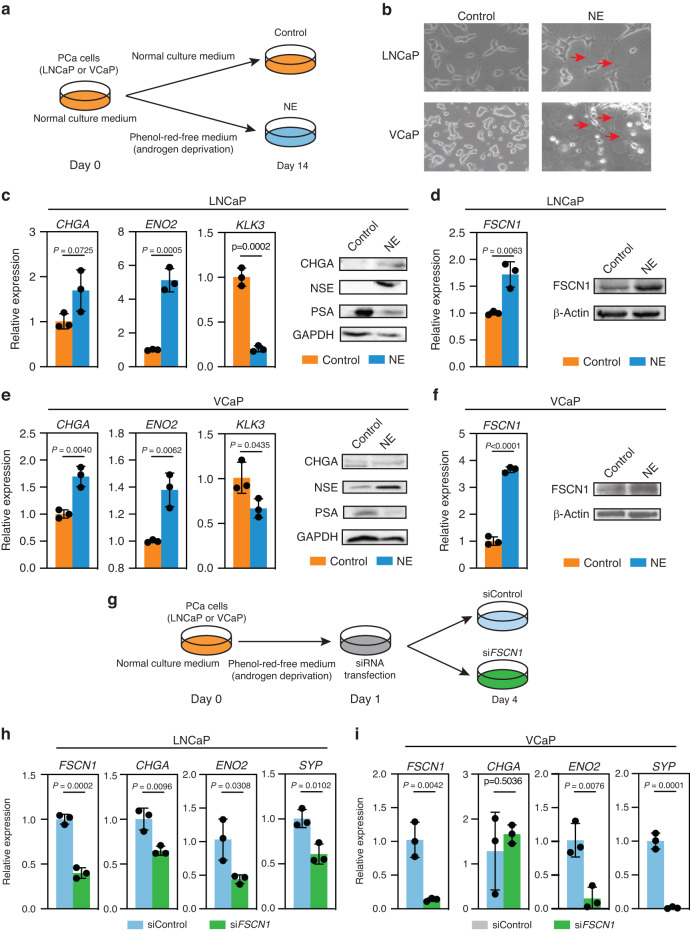
Androgen deprivation induces a neuroendocrine-like phenotype and triggers *FSCN1* expression. **a** Schematic representation of NE transdifferentiation using LNCaP and VCaP cells cultured with the charcoal-stripped medium in androgen-deprivation conditions for 14 days (NE), compared with LNCaP and VCaP cultured with the normal medium in androgen-sensitive conditions (Control). **b** Representative phase-contrast microscopic images of the morphology of LNCaP and VCaP cells cultured for 14 days in normal medium (LNCaP and VCaP) or cultured in charcoal-stripped medium (NE). Arrowheads indicate neurite-like cell structures. **c**, **e** LNCaP (**c**) and VCaP (**e**) cells were cultured for 14 days in a charcoal-stripped medium to induce a neuroendocrine-like phenotype (NE). RT-PCR showing expression of neuroendocrine markers *Chromogranin A (CHGA)* and *Enolase-2 (ENO2)*. *KLK3* expression is a positive control of androgen activity. Error bars indicate *n* = 3, mean +/− s.d. Statistical significance was determined by a two-tailed unpaired Student *t* test. Western blot validation of CHGA and NSE protein expression in NE cells and PSA protein expression in Control cells. GAPDH was used as a loading control. **d**, **f** RT-PCR evaluating expression levels of *FSCN1* in LNCaP (**d**) and in VCaP (**f**), respectively. Error bars indicate *n* = 3, mean +/− s.d. Statistical significance was determined by a two-tailed unpaired Student *t* test. Western blot validation of FSCN1 expression cells. β-actin was used as a loading control. **g** Schematic representation of the timeline for siRNA transfection of LNCaP and VCaP cells in androgen-deprivation culture conditions. **h**, **i** RT-PCR evaluating expression levels of *FSCN1, CHGA, ENO2* and *Synaptophysin (SYP)* in LNCaP cells (**h**) and VCaP cells (**i**) with si*FSCN1* in comparison with siControl. Error bars indicate *n* = 3, mean +/− s.d. Statistical significance was determined by a two-tailed unpaired Student *t* test.

Lineage plasticity in PCa has been linked to neuroendocrine differentiation involving transdifferentiation of prostate adenocarcinoma cells. The underlying mechanisms have been studied using androgen-deprivation cell models [47]. To further demonstrate that *FSCN1* expression is associated with an NEPC phenotype, we took advantage of a cell model consisting of an androgen-depleted culture of LNCaP and VCaP cell lines [47]. Using a phenol-red-free medium and a charcoal-depleted serum to remove androgens, we mimicked androgen deprivation in culture (Fig. 4a). Compared with unstripped serum conditions (LNCaP or VCaP), cells cultured for 14–21 days in androgen-deprivation conditions (LNCaP-NE or VCaP-NE) gradually developed elongated cytoplasmic protrusions, neurite-like structures, consistent with previous reports (Fig. 4b) [48]. Importantly, LNCaP-NE and VCaP-NE cells showed increased expression of NEPC markers, such as *CHGA, ENO2, SYP*, and the NE regulators, the *neural POU-domain transcription factor BRN2*, and the *SRY-related HMG-box gene 2 (SOX2)*, and showed a strong decrease in *KLK3* (Fig. 4c, e and Supplementary Fig. S3a, b). Measured after 14 days of androgen-deprivation culture, LNCaP-NE and VCaP-NE showed a remarkable increase in *FSCN1* expression at the transcript and protein levels (Fig. 4d, f). Because FSCN1 has been described as an important protein for invadopodia formation [48, 49], which are crucial for tumour cell invasion in cancer progression, we evaluated the ability of LNCaP cells to form invadopodia under androgen-deprivation conditions (Supplementary Fig. S3c, d). The presence of invadopodia was confirmed by culturing cells on top of a fluorescently conjugated matrix (fluorescein gelatin), staining cells for F-actin and examining colocalization between F-actin puncta and degradation of fluorescent gelatin (black regions) [49]. After 6 h, matrix degradation was identified with the appearance of fluorescence-negative areas (black holes in Supplementary Fig. S3c, d), F-actin and nucleus labelling, using phalloidin and Hoechst dyes, respectively, revealed LNCaP invadopodia formation in androgen-deprivation culture conditions [50]. Using antibodies against FSCN1, we detected the FSCN1 protein -as expected- in the matrix degradation area corresponding to the invadopodia [22].

Next, we examined whether the expression of *FSCN1* is associated with NE marker expression during the emergence of NE phenotype in LNCaP and VCaP cells. As shown in Fig. 4g, si*FSCN1* were transfected on day 1 after androgen-deprivation culture (i.e., at the beginning of NE differentiation induction), and then RNA analyses were performed on day 4 post-NE induction. In Fig. 4h, i, we showed that knocking down *FSCN1* expression by 60% in LNCaP and 90% in VCaP, reduced NE marker expression compared with the siControl. Moreover, we also found the expression of FSCN1, at mRNA and protein levels, in NCI-H660 cells, a unique and typical NEPC cell line (Supplementary Fig. S4).

Taken together, these results suggested a potential role of *FSCN1* in neuroendocrine transdifferentiation, and FSCN1 may be a new NEPC marker.

#### FSCN1 knockdown reduced tumour growth of AR-negative cells

**Fig. 5:**
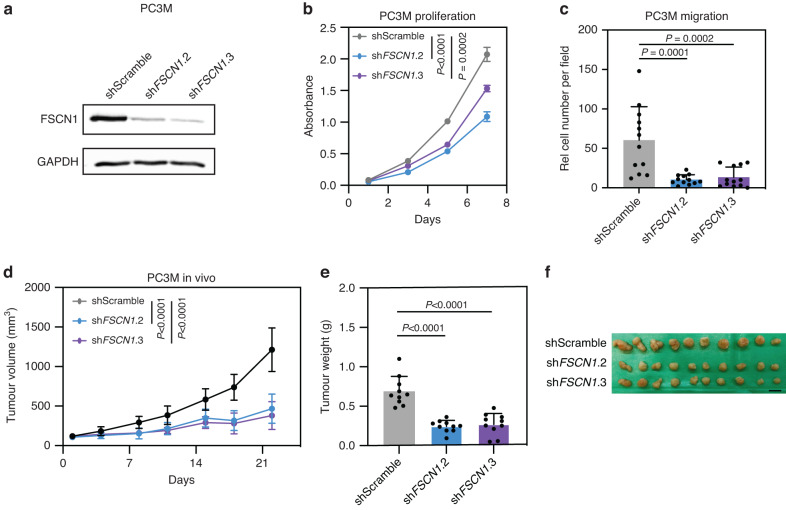
FSCN1 loss affects prostate cancer cell growth in vitro and in vivo. **a** FSCN1 protein expression was assessed by western blotting in PC3M cells expressing shScramble, shFSCN1.2, and shFSCN1.3, respectively. GAPDH was used as a loading control. **b** Cell proliferation of PCM3 cells expressing shScramble, shFSCN1.2, and shFSCN1.3 was assessed by MTT assay. **c** Cell migration of PCM3 cells expressing shScramble, shFSCN1.2, and shFSCN1.3 was assessed by Transwell assay. Relative cell numbers of cells per field are shown. **d** PC3M cells expressing shScramble, shFSCN1.2, or shFSCN1.3 were implanted subcutaneously, and tumour growth was assessed by biweekly measurements. **e** Tumour weight of PC3M tumours expressing shScramble, shFSCN1.2, or shFSCN1.3 at the endpoint. **f** Images of excised tumours at the endpoint.

It has been shown that FSCN1 expression correlates with poor clinical outcomes and shorter survival across different cancer types [51]. Thus, we next studied the role of FSCN1 in AR-negative PCa cells. Among the possible cellular model, NCI-H660 is a cell line derived from human NEPC. However, this PCa cell line shows extremely slow growth kinetics, limiting its utilisation in downstream functional analysis. We choose the PC3M cells, derived from PC3, which express *FSCN1*, as shown in Supplementary Fig. S4. Moreover, these cells are AR-independent (AR- and PSA-) with characteristics of prostatic small cell neuroendocrine carcinoma [52, 53]. We established PC3M cell lines expressing short hairpin RNA control (shScramble), or short hairpin RNA targeting specifically *FSCN1* (shFSCN1.2 and shFSCN1.3). Of note, shFSCN1.2 and shFSCN1.3 inhibited the expression FSCN1 efficiently (Fig. 5a). Importantly, *FSCN1* knockdown significantly decreased proliferation and cell migration of PC3M cells in vitro (Fig. 5b, c). We next assessed the functional impact of *FSCN1* knockdown on tumour growth in vivo. PC3M cells expressing either shScramble, shFSCN1.2 or shFSCN1.3 were subcutaneously implanted into NOD-SCID mice. We could find that compared to shControl tumours, the tumour growth of shFSCN1.2 and shFSCN1.3 was delayed (Fig. 5d). In addition, tumours formed by PC3M cells expressing shFSCN1.2 and shFSCN1.3 are significantly smaller than those formed with PC3M cells expression shScramble (Fig. 5e, f). Taken together, these in vitro and in vivo studies suggest that *FSCN1* knockdown could inhibit the proliferation and migration of neuroendocrine-like cells, leading to tumour growth delay.

### AR binds directly to the FSCN1 regulatory sequence

**Fig. 6:**
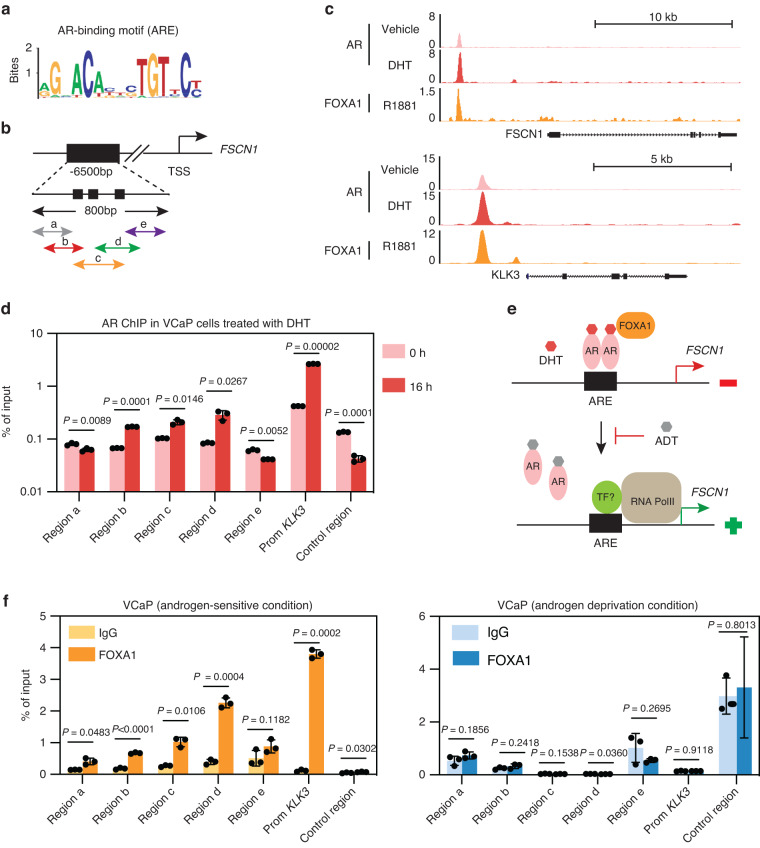
AR protein binds to cis-elements of the *FSCN1* gene. **a** Schema showing the AR-binding motif (ARE) obtained from JASPAR database. **b** Genomic location of the potential AREs on the *FSCN1* gene (TSS: transcription start site). Location of the qPCR-amplified fragments (a–e) overlapping potential AREs in an 800 bp domain (little black boxes). **c** UCSC genome browser representations showing AR and FOXA1 binding events on *FSCN1* and *KLK3* loci in VCaP cells. ChIP-seq profiles indicate AR enrichment (pink and red) and FOXA1 enrichment (orange) on *FSCN1* and *KLK3* gene loci in the vehicle (GSM1328945 in pink), DHT-stimulated (GSM1328952 in red) or R1881-stimulated (GSM1354839 in orange) VCaP cells. **d** ChIP-qPCR assay confirmation of AR binding on the *FSCN1* regulatory region in DHT-stimulated VCaP cells (16 h), compared with untreated cells (0 h), using either anti-AR or anti-IgG antibodies. The results are expressed in % of input for each fragment a to e. *KLK3* promoter (Prom KLK3) was used as a positive control for the DHT-stimulation experiment, and an irrelevant region (Control Region) was used as a negative control. Experiments were performed with n = 3 biologically independent samples. Data were presented as mean +/− s.d. Statistical significance was determined by a two-tailed unpaired Student *t* test. **e** Schema illustrating AR signalling-mediated regulation of *FSCN1* gene transcription in prostate cancer. AR Androgen receptor, DHT dihydrotestosterone, FOXA1 forkhead box A1, ADT androgen-deprivation therapy/AR antagonists, RNA PolII RNA polymerase, TF transcription factors. **f** ChIP-qPCR assays of FOXA1 binding on the *FSCN1* regulatory region a–e in VCaP cells cultured in androgen-sensitive (left panel) or androgen-deprivation (right panel) conditions using either anti-FOXA1 or anti-IgG antibodies. The results are presented in % of input. The *KLK3* promoter (Prom KLK3) was used as a positive control for the DHT-stimulation experiment. An irrelevant region (Control region) was used as a negative control. Experiments were performed with *n* = 3 biologically independent samples. Data were presented as mean +/− s.d. Statistical significance was determined by a two-tailed unpaired Student *t* test.

Given that the expression of *FSCN1* was found in AR-negative PCa cells (Fig. 1a), we examined whether the *FSCN1* gene expression was downregulated by a direct AR-mediated mechanism in PCa cells. First, we took advantage of an AR-negative cell line, the PC3c-T1E4 model, which highly expresses the *FSCN1* gene, to transiently overexpress AR and measure *FSCN1* expression variations (Supplementary Fig. S1a, c). Using transient transfection of an AR expression vector, we showed that AR expression significantly decreased *FSCN1* expression at the transcriptional levels (Supplementary Fig. S1c). Then, we hypothesised that AR could transcriptionally repress *FSCN1* in AR-positive VCaP and LNCaP cells by directly binding to the androgen response element (ARE) on the *FSCN1* gene. Because AR has been described both as a transcriptional activator and repressor [54], we looked for putative AREs in the *FSCN1* regulatory regions. We used transcription factor prediction software for transcription factor binding site identification (HOMER) (Fig. 6a) and analysed publicly available ChIP-sequencing datasets (ChIP-seq) for AR binding to identify and localise AR binding 6500 bp upstream of the *FSCN1* gene. Using datasets obtained with normal prostate tissues, prostate tumour samples and androgen-dependent cell lines such as VCaP and LNCaP cells (Fig. 6b, c and Supplementary Fig. S5), we identified a unique and major AR-binding peak in tumours and cell lines corresponding to AREs, located 6500 bp upstream of the *FSCN1* transcriptional start site. The *KLK3* gene, known to be regulated by AR through its direct and specific DNA binding, was used as a control in this analysis (Fig. 6c and Supplementary Fig. S6).

To test whether AR directly binds to the identified AREs of the *FSCN1* gene, we performed ChIP-qPCR assays with VCaP cells, untreated or DHT-stimulated for 16 h. Using five amplification fragments covering the potential AR-binding regions (Fig. 6b), we found that AR binding was enriched in three DNA fragments corresponding to potential AREs (Fig. 6d). The promoter of the *KLK3* gene was used as a positive control (Fig. 6d). These data are consistent with a mechanism (Fig. 6e) whereby AR acts as a direct transcriptional repressor of *FSCN1* expression, potentially associated with corepressors, and therefore, therapies based on androgen deprivation may reduce *FSCN1* transcriptional repression, hence resulting in its expression.

Among the transcription factors known to contribute to the AR transcription activation or repression properties, we sought to examine the potential role of the FOXA1 (forkhead box A1) transcription factor (Fig. 6e), which has been shown to play a key role in AR-mediated gene regulation. FOXA1 is known to interact with AR and co-occupy the chromatin binding [55]. To further investigate the potential role of FOXA1 in *FSCN1* gene transcriptional repression, we analysed public ChIP-seq datasets for FOXA1 binding in androgen-dependent VCaP and LNCaP cells (Fig. 6c and Supplementary Fig. S7). We identified a FOXA1 binding element in the same area as the identified AREs within the *FSCN1* gene. To further confirm FOXA1 binding to the *FSCN1* gene regulation region, we performed CHIP-qPCR experiments on VCaP cells cultured with and without a charcoal-stripped medium. Results showed enrichment in FOXA1 binding sites at ARE fragments when a regular medium was used (Fig. 6f, left panel). The *KLK3* promoter was used as the positive control (Fig. 6c and Supplementary Fig. S8). In contrast, both AR and FOXA1 binding were drastically reduced in a hormone-depleted medium (Fig. 6f, right panel). These results suggest that at least AR and FOXA1 regulate *FSCN1* expression in an androgen-dependent mechanism.

Finally, to assess the enhancer activity of the *FSCN1* gene in NEPC, we took advantage of the genome-wide H3K27 acetylation analyses performed by Baca et al. [34], in NEPC and prostate adenocarcinoma (PRAD) LuCap patient-derived xenografts (PDXs). In Supplementary Fig. S9, the *FSCN1* gene shows an increase in H3K27 acetylation in NEPC compared with PRAD samples, suggesting increased enhancer activity in NEPC. As controls, the *CHGA* and *KLK3* genes, specifically expressed in NEPC and PRAD, showed respectively an increase and a decrease in H3K27 acetylation, as expected. This pattern suggests that *FSCN1* gene may be regulated by a neuroendocrine transcriptional programme.

## Discussion

NEPC is a highly aggressive form of PCa that is increasing in incidence in association with the development of resistance to AR pathway inhibitors [3, 56]. However, the molecular mechanisms associated with NEPC development and invasiveness are still poorly understood. The present study uncovers a previously unknown role for the FSCN1 protein in NEPC. FSCN1 is an actin-bundling protein that plays a key role in actin stability in the invadopodia [49, 57]. By increasing membrane protrusions (filopodia and invadopodia), facilitating focal adhesion turnover and regulating nuclear organisation, FSCN1 promotes the physical translocation of metastatic cancer cells [57]. Importantly, FSCN1 overexpression has been strongly associated with poor prognosis and metastatic progression across different cancer types [24–27, 57]. Here, we showed that, in addition to being significantly and specifically overexpressed in clinical NEPC samples, FSCN1 knockdown expression, particularly at the beginning of cell culture in androgen-deprivation conditions, was able to decrease the expression of NE markers significantly. This suggests that FSCN1 plays an important role in the NE marker expression and phenotype. Moreover, FSCN1 knockdown enhanced the ability of cells to migrate, as assessed by cell migration assays in vitro. Therefore, our study connects NE differentiation with invadopodia formation. In vivo, FSCN1 knockdown inhibits tumour growth in mice. This finding, together with the observations of its effect on NE markers expression, suggests that FSCN1 might act as an important mediator in NEPC development.

NEPC can emerge after therapeutic pressure through a process of tumour cell transdifferentiation from preexisting adenocarcinoma and very rarely de novo [58]. Indeed, the improved potency and specificity of ADT have led to an increased prevalence of NEPC [59, 60]. Because NEPC features androgen deprivation and because the loss of AR expression has been described to enable NE differentiation in androgen-dependent cell lines, a question arises as to whether AR is able to repress the expression of NEPC genes and *FSCN1* in particular. Here, we provide compelling evidence that *FSCN1* is a direct androgen-repressed gene. AR is a hormone transcription factor, best known as an activator. It is associated with coactivators and chromatin modifiers to induce transcription of AR target genes, which are defined by direct AR binding to AREs at their regulatory genomic regions [61].

In our study, analyses of publicly available ChIP-seq datasets and ChIP-qPCR experiments revealed a direct AR binding in androgen-dependent conditions, where *FSCN1* expression is repressed. The AR-binding sites were identified 6.5 kb downstream from the transcription start site, and our results suggest that, in androgen-dependent conditions, AR acts as an *FSCN1* gene repressor, possibly with corepressors. In contrast, a decrease or absence of AR binding was observed in androgen-deprivation conditions. One of the possible AR corepressors may be FOXA1, a pioneering factor which makes chromatin accessible to AR, known to facilitate AR recruitment to target gene AREs [62]. Moreover, AR is known to bind to enhancer regions rather than to the promoter, recruiting FOXA1, thus activating enhancers [63]. Based on these reports, we took advantage of recently published ChIP-seq-FOXA1 datasets, to identify a peak in ChIP-seq-FOXA1 located in the same AR-binding genomic region and confirmed that FOXA1 binds the same DNA fragments as AR in androgen-dependent conditions. Therefore, FOXA1 may act as an AR corepressor and thus downregulates *FSCN1* gene expression. Finally, because the H3K27ac mark has been reported to be a reliable predictor of enhancer activity, we showed that the H3K27ac profile of the *FSCN1* gene in at least five NEPC samples revealed strong activity, similar to *CHGA*, an NE marker. In contrast, in five primary tumours (i.e. PRAD), only *KLK3* revealed strong H3K27ac activity.

Finally, NEPC is mostly defined as a subset of CRPC with a loss of AR expression or activity. Clinically, patients present low PSA levels with high metastatic burden in soft tissues [64]. Since AR is best known as a transcription activator, efforts have been focused on AR-activated genes and ADT is the standard of care for PCa patients. However, the mechanisms by which the AR directly influences the induction of the NEPC phenotype and the expression of specific NE markers may involve key AR-regulated genes, and particularly AR-repressed genes, which could be re-expressed in absence of AR or AR-pathways [54]. For example, AR have been shown to directly repress transcription of a master neural transcription factor BRN2 and the reprogramming transcription factor SOX2, both playing a significant role in the progression of PCa to NEPC [65, 66]. Therefore *FSCN1* gene could be part of the AR-repressed genes, such as *BRN2*, *SOX2*, *CCND1* (*Cyclin-D1*), *c-MET* (Hepatocyte growth/scatter factor receptor), which are re-expressed in the aggressive AR-independent form of the disease and involved in emergence or maintenance of the NEPC phenotype. Since *FSCN1* overexpression has been correlated with poor clinical outcomes and shorter survival across different cancer types, FSCN1 has been suggested as a therapeutic target for blocking migration, invasion and metastasis, encouraging FSCN1 inhibitors identification [57]. In addition, recent findings suggest that FSCN1 may be a useful druggable target for controlling adrenocortical carcinoma, a rare disease [67]. Currently, some of these FSCN1 inhibitors, which block the actin-bundling activity of FSCN1, are in clinical development in early-phase trials (NCT03199586, NCT05023486).

In conclusion, given that NEPC has a poor prognosis and very limited therapeutic options, there is an urgent need to control NE lineage transdifferentiation and to develop novel therapeutic approaches that can extend the clinical response to ADT. Interestingly, AR-repressed genes represent promising markers and targets for diagnosis and therapeutic interventions in NEPC progression. Our data suggest that FSCN1 may be considered as a new actor (i.e. mediator or “facilitator”), restricted to NEPC. Although *FSCN1* promotes the progression of many human cancers, it has not yet been approved as a biomarker in clinical practice. Therefore, FSCN1 now needs to be studied in a well-designed prospective study to assess its independent value as a new biomarker. Targeting FSCN1 might be a promising approach to treat NEPC.

### Supplementary information

Supplementary Figure legends

Supplementary Figure S1

Supplementary Figure S2

Supplementary Figure S3

Supplementary Figure S4

Supplementary Figure S5

Supplementary Figure S6

Supplementary Figure S7

Supplementary Figure S8

Supplementary Figure S9

Supplementary Table S1

Supplementary Table S2

Supplementary Table S3

## Data Availability

All the materials used to produce the data in this study are available from the corresponding authors upon reasonable request.
