# Supplementary material for: Fascin-1 expression is associated with neuroendocrine prostate cancer and directly suppressed by androgen receptor
Source: Br J Cancer. 2023 Oct 24;129(12):1903–14. doi: 10.1038/s41416-023-02449-x (PMC10703930; doi:10.1038/s41416-023-02449-x)
Supplement: Supplementary file 1 — Supplementary Figure legends [file 41416_2023_2449_MOESM1_ESM.docx]

**Supplementary figure legends, figures and tables**

**Fig. S1 *FSCN1* expression in an AR-negative PC3c-TIE4 model. a** RT-PCR showing *FSCN1* expression in stable ERG-T1E4-overexpression in PC3c cells compared with pcDNA control cells. The mean +/- s.d. of duplicate experiments run in triplicate. Statistical significance was determined by two-tailed unpaired Student *t*-test. Western blot validating the expression of ERG-T1E4 and FSCN1 in ERG-T1E4 and pcDNA control cells. Vinculin was used as loading control. **b** Gene expression of *FSCN1* in prostate cancer tissue samples (PCa, n=52) and in normal prostatic samples adjacent to tumor (N-PCa, n=20) (left panel), in TMPRSS2:ERG Fusion-positive (n=32) and Fusion-negative (n=20) prostate cancer samples (central panel) were evaluated using RT-qPCR in triplicate. Two independent experiments were performed. Non-significant differences were determined by two-tailed Mann–Whitney test (p=0.7982 and p=0.3454 respectively). Scatter plot showing relative *FSCN1* mRNA expression and its corresponding ERG mRNA expression in primary PCa samples (n=52). Spearman r=0.2025, p=0.1499 (non-significant), two-tailed non-parametric Spearman correlation test with alpha=0.05. **c** RT-PCR showing *FSCN1* downregulation following AR overexpression using PC3c-T1E4 cells transfected with PSG5-AR or PSG5 vector control. The mean +/- s.d. of duplicate experiments run in triplicate. Statistical significance was determined by two-tailed unpaired Student *t*-test. Western blot validation of AR and FSCN1 expression 48 h post-transfection in PC3c-T1E4 cells. Vinculin was used as loading control.

**Fig. S2 FSCN1 expression in metastatic PCa samples is restricted to blood vessels, osteoblasts and osteocytes. a-b** Immunohistochemical (IHC) staining was performed on bone (**a**) and lymph node (**b**) metastasis PCa samples, using two distinct antibodies against FSCN1 (ab49815 Abcam and FCN01 55K-2 ThermoFisher). BM: bone matrix, OB: osteoblast, Ocy: osteocyte, LT: lymphatic tissue, T : Tumor. Scale bar = 50 µm.

**Fig S3 NEPC expression markers.** **(a-b)** LNCaP **(a)** and VCaP **(b)** cells are cultured 14 days with charcoal-depleted medium to induce neuroendocrine-like phenotype LNCaP-NE and VCaP-NE. RT-PCR showing expression of neuroendocrine markers *Synaptophysin* (SYP), *SRY-related HMG-box gene 2* (SOX2) and *neural POU-domain* transcription factor (BRN2). Error bars indicate n=3, mean +/- s.d. Statistical significance was determined by two-tailed unpaired Student t test. **c** LNCaP are able to form invadopodia and degrade fluorescent matrix. Representative images of LNCaP cells seeded on fluorescein-conjugate gelatin-coated (Invitrogen G13187) coverslips for 3 hours. After fixation, F-actin filaments were labeled with Alexa Fluor® 555 Phalloidin (red) (Cell Signaling 8953S), FSCN1 was labelled in pink by using anti-FSCN1 (abcam126772, Abcam Epitomics, Cambridge, UK) at 1/100, nucleus were labelled by Hoechst. The active invadopodia were identified thanks to degradation area in fluorescent-gelatin (absence of fluorescence in dark regions of the matrix). **d** Schematic diagram of fluorescent matrix degradation assay. Black lines correspond to the xy or xz planes: xy projections presenting the cell and xz projections showing longitudinal cut through the cell at the level of the protrusions inserted into the underlying gelatin (green). In protrusions, F-actin (left panel) and FSCN1 (right panel) are visible in red and pink respectively.

**Fig S4 *FSCN1* expression in NCI-H660 cells, a unique and typical NEPC cell line. a** RT-PCR evaluating expression levels of *FSCN1* from LNCaP, VCaP, NCI-H660 and PC3M cell lines. **b** Western blot validating expression FSCN1 proteins in NCI-H660 and PC3M cell lines. GAPDH was used as loading control. Error bars indicate, mean +/- s.d. Statistical significance was determined by one-way ANOVA with a Dunnett multiple comparison test (n=3).

**Fig S5 UCSC genome browser representations showing AR binding events on *FSCN1* locus in 7 cell lines and 20 patient prostate tissues.** ChIP-seq profiles indicating AR enrichment on *FSCN1* locus in vehicle (black), AR activator-treated (red), AR inhibitor-treated (blue) or both AR inhibitor and activator-treated (green) in VCaP, LNCaP, 22RV1, C4-2B, CWR22Pc, R1-AD1 and VCS2 cells, and in patient prostate normal (n=8) and tumor (n=12) tissues. Different datasets were used, with different AR activators, DHT or R1881, and different AR inhibitors, bicalutamide or enzalutamide. H3K27Ac, H3K4me1 (enhancer mark), H3K4me3 (promoter mark), and DNase-seq profiles from the human ENCODE project are also shown.

**Fig S6 UCSC genome browser representations showing AR binding events on *KLK3* locus in 7 cell lines and 20 patient prostate tissues.** ChIP-seq profiles indicating AR enrichment on *KLK3* locus in vehicle (black), AR activator-treated (red), AR inhibitor-treated (blue) or both AR inhibitor and activator-treated (green) in VCaP, LNCaP, 22RV1, C4-2B, CWR22Pc, R1-AD1 and VCS2 cells, and in patient prostate normal (n=8) and tumor (n=12) tissues. Different datasets were used, with different AR activators, DHT or R1881, and different AR inhibitors, bicalutamide or enzalutamide. H3K27ac, H3K4me1 (enhancer mark), H3K4me3 (promoter mark), and DNase-seq profiles from the human ENCODE project are also shown.

**Fig S7 UCSC genome browser representations showing AR and FOXA1 binding events on *FSCN1* locus in 2 cell lines and 29 prostate cancer PDXs.**  ChIP-seq profiles indicating AR (red) and FOXA1 (black) enrichment on *FSCN1* locus in LNCaP and VCaP cells, and in adenocarcinoma (n=23) or neuroendocrine (n=6) prostate cancer PDX. Different datasets were used, but all ChIP-seq experiments were performed using cells AR activator-treated (DHT or R1881). H3K27ac, H3K4me1 (enhancer mark), H3K4me3 (promoter mark), and DNase-seq profiles from the human ENCODE project are also shown.

**Fig S8 UCSC genome browser representations showing AR and FOXA1 binding events on *KLK3* locus in 2 cell lines and 29 prostate cancer PDXs.** ChIP-seq profiles indicating AR (red) and FOXA1 (black) enrichment on *KLK3* locus in LNCaP and VCaP cells, and in adenocarcinoma (n=23) or neuroendocrine (n=6) prostate cancer PDX. Different datasets were used, but all ChIP-seq experiments were performed using cells AR activator-treated (DHT or R1881). H3K27ac, H3K4me1 (enhancer mark), H3K4me3 (promoter mark), and DNase-seq profiles from the human ENCODE project are also shown.

**Fig. S9 Enhancer activity of the *FSCN1* gene in neuroendocrine prostate cancer.** Analyses of the enhancer-associated histone modification H3K27-acetylated (H3K27ac) profiles at the *FSCN1*, *KLK3* and *CHGA* genes in five prostate cancer adenocarcinoma (PRAD) and five neuroendocrine prostate cancer (NEPC) patient-derived xenografts (PDXs) from Baca et al. 2021. The PDX sample numbers are LuCaP-70, -78, -78CR, -189.4 and -77 for PRAD samples and LuCaP-93, -173.1, -49, -145.1 and -145.2 for NEPC samples described in [34].

**Supp Table 1 : Quantitative PCR primers**

**Supp Table 2 : Chromatin-Immunoprecipitation-quantitative PCR primers**

**Supp Table 3 : NEPC patients**
