# Supplementary figures and images for: Fascin-1 expression is associated with neuroendocrine prostate cancer and directly suppressed by androgen receptor
Source: Br J Cancer. 2023 Oct 24;129(12):1903–14. doi: 10.1038/s41416-023-02449-x (PMC10703930; doi:10.1038/s41416-023-02449-x)

# Supplementary Figure S1

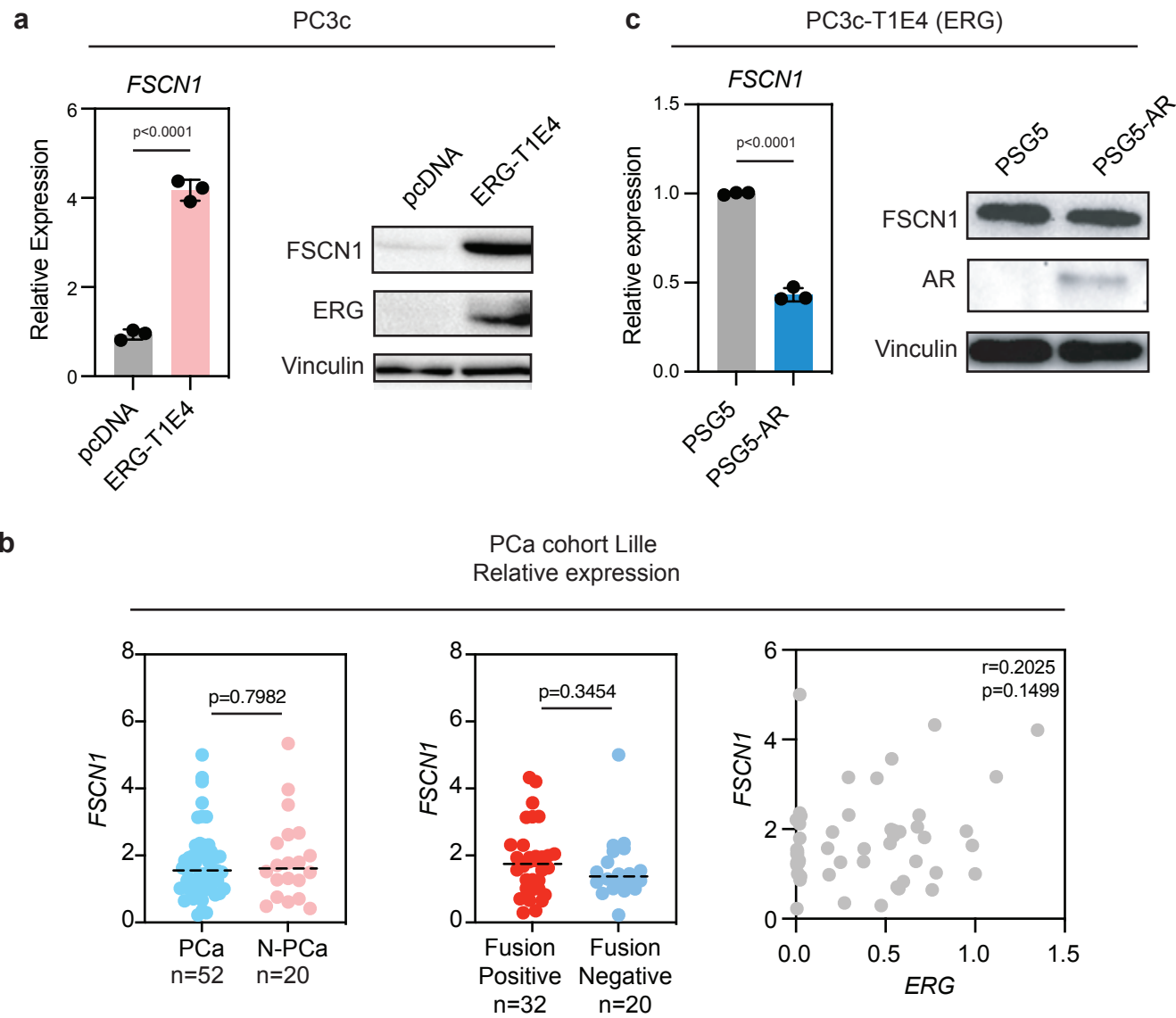

Supplement: Supplementary file 2 — Supplementary Figure S1 [file 41416_2023_2449_MOESM2_ESM.pdf]

# Supplementary Figure S3

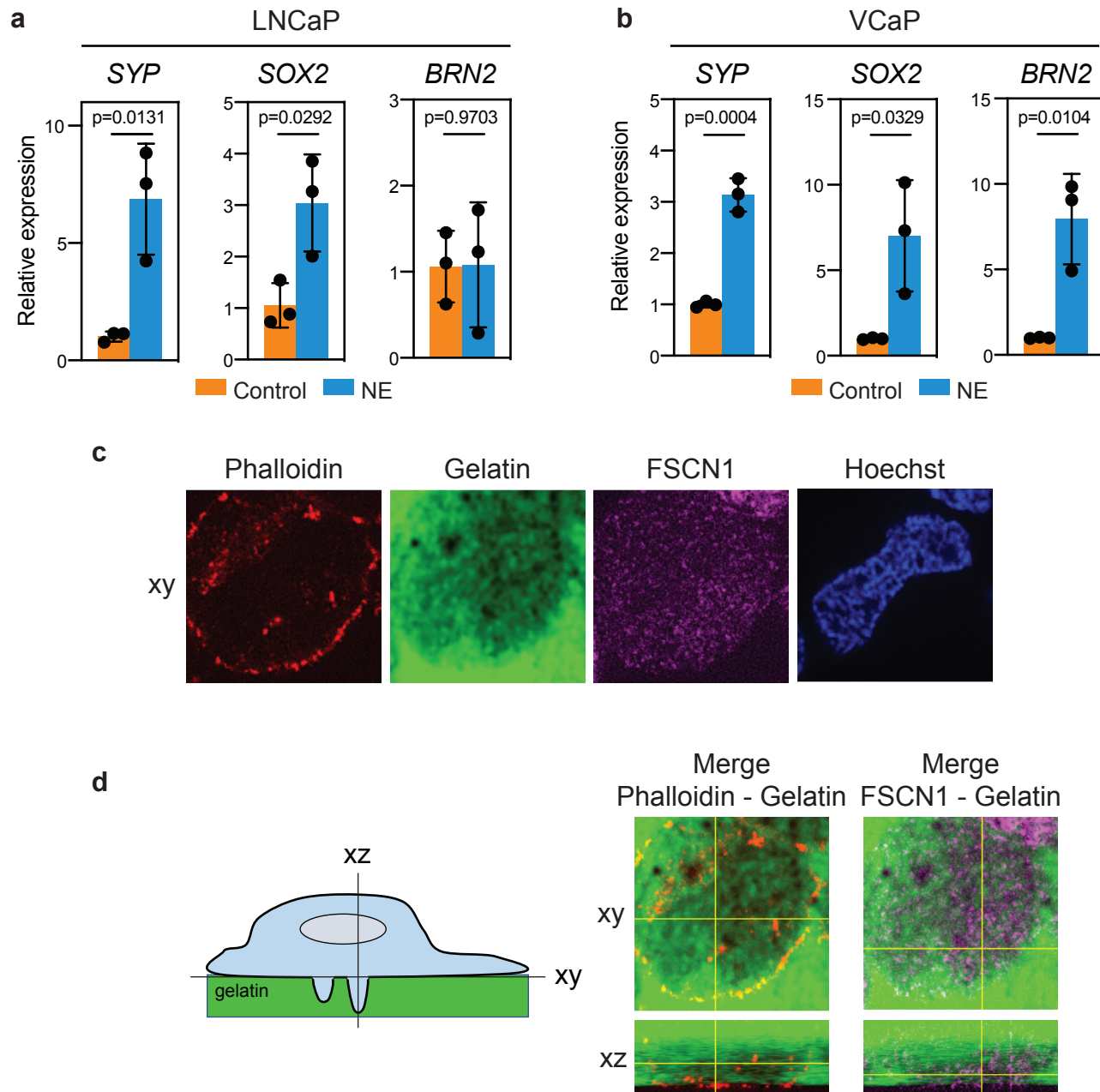

Supplement: Supplementary file 4 — Supplementary Figure S3 [file 41416_2023_2449_MOESM4_ESM.pdf]

# Supplementary Figure S4

**a**

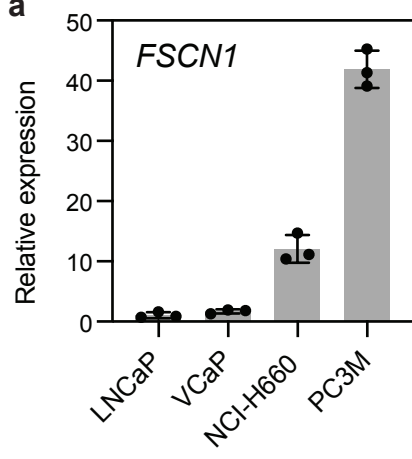

**b**

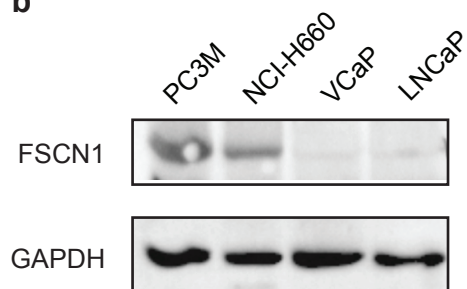

Supplement: Supplementary file 5 — Supplementary Figure S4 [file 41416_2023_2449_MOESM5_ESM.pdf]

# Supplementary Figure S5

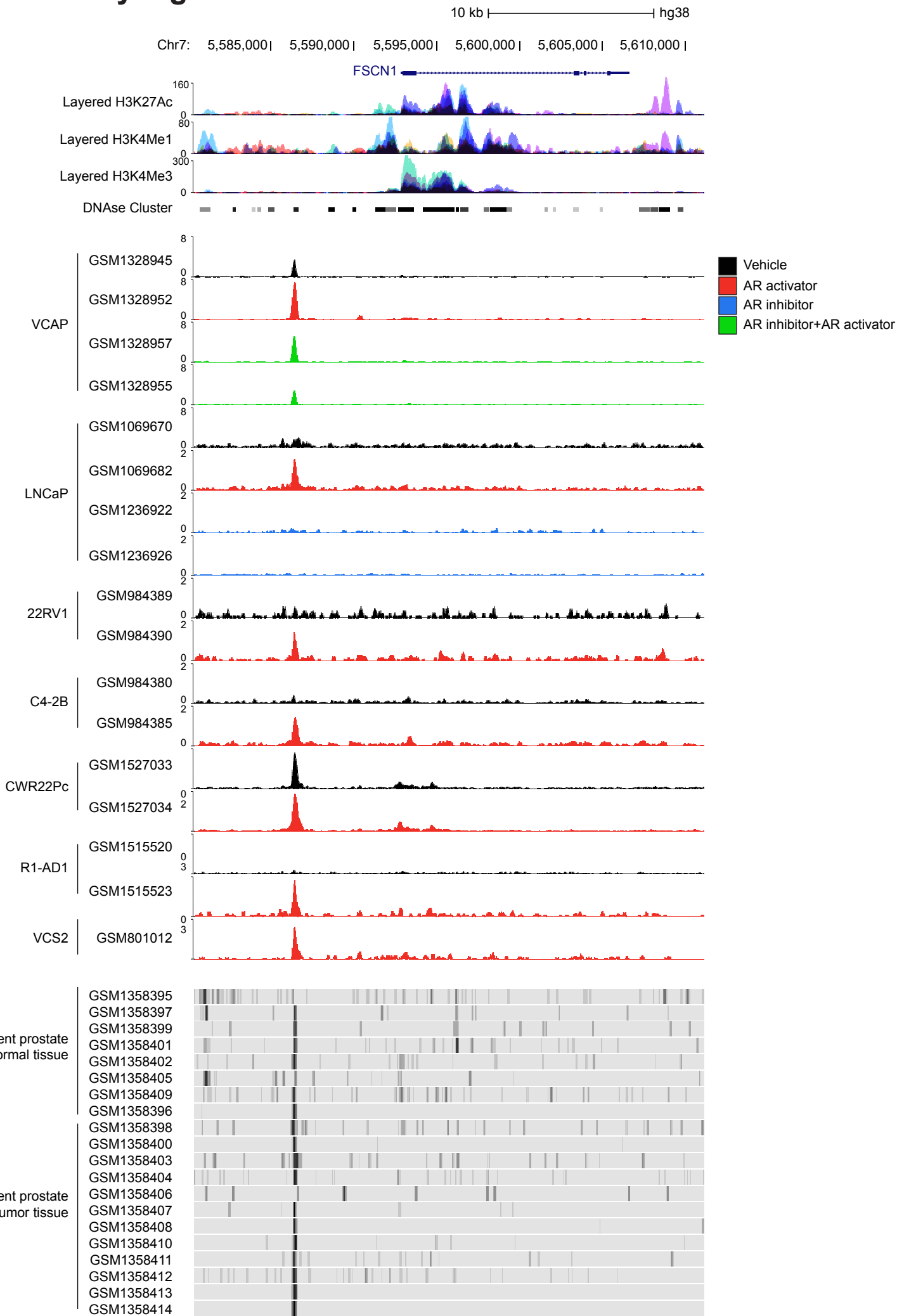

Supplement: Supplementary file 6 — Supplementary Figure S5 [file 41416_2023_2449_MOESM6_ESM.pdf]

# Supplementary Figure S6

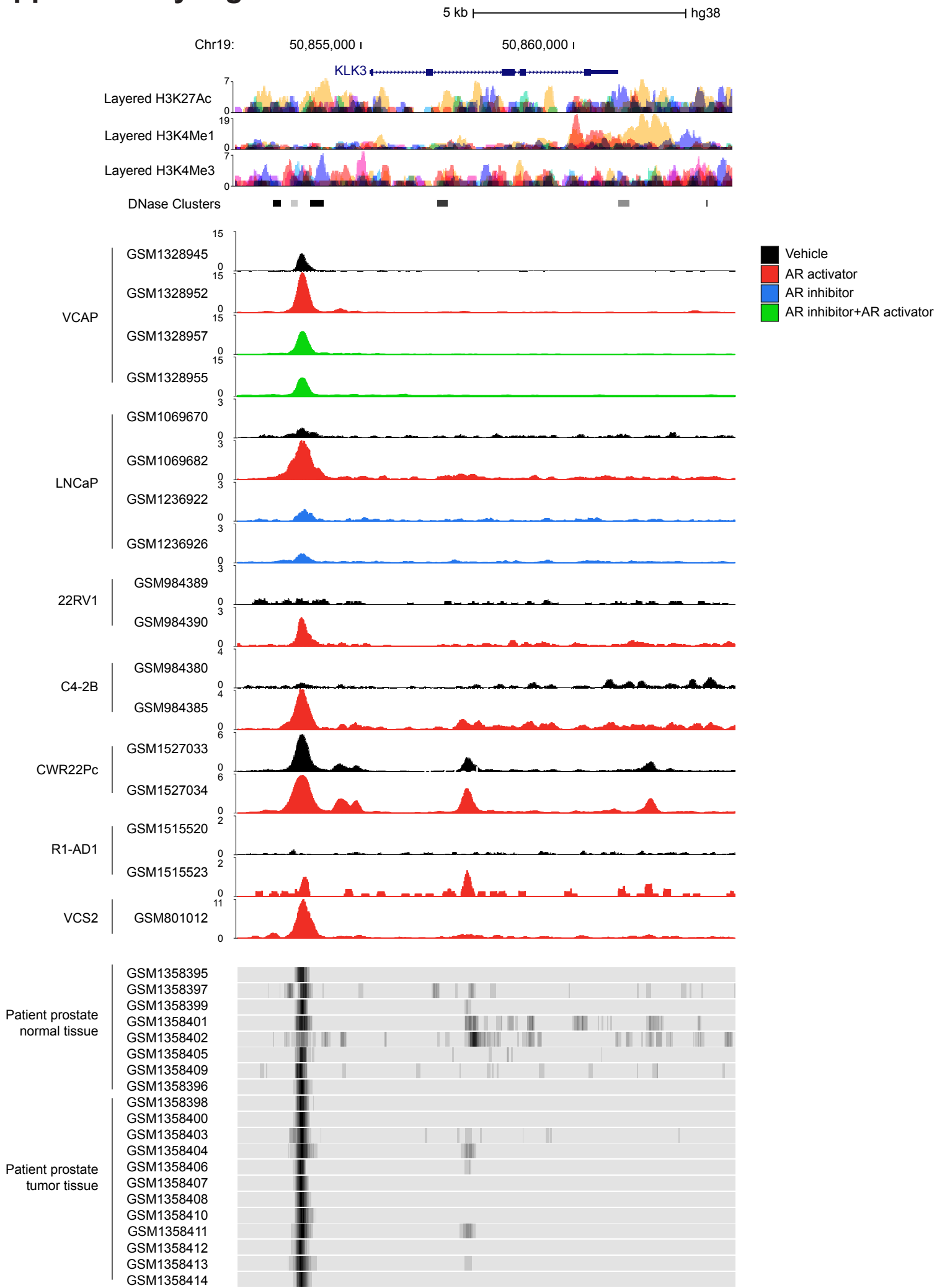

Supplement: Supplementary file 7 — Supplementary Figure S6 [file 41416_2023_2449_MOESM7_ESM.pdf]

# Supplementary Figure S7

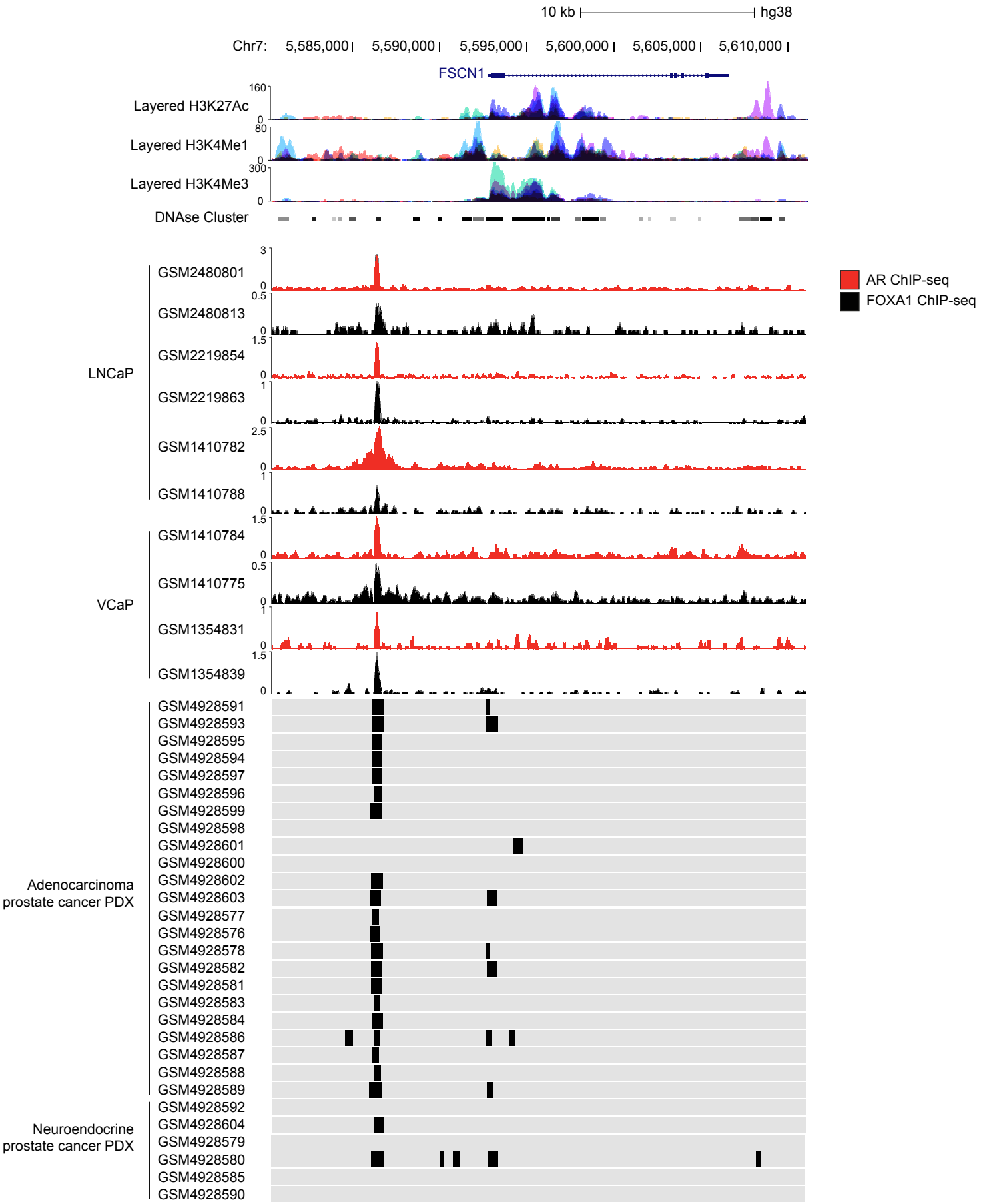

Supplement: Supplementary file 8 — Supplementary Figure S7 [file 41416_2023_2449_MOESM8_ESM.pdf]

Figure Supplementary Figure S8

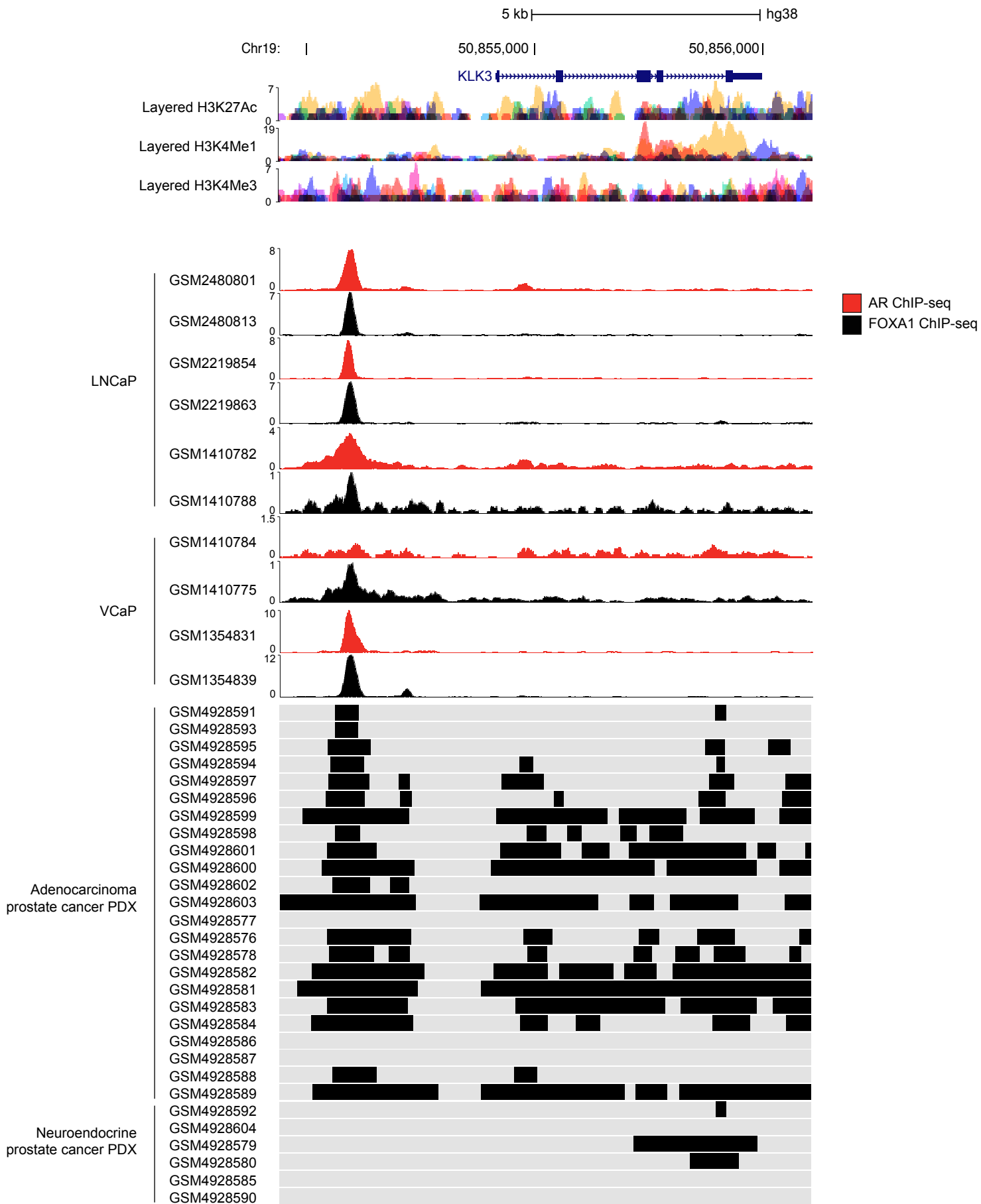

Supplement: Supplementary file 9 — Supplementary Figure S8 [file 41416_2023_2449_MOESM9_ESM.pdf]

# Supplementary Figure S9

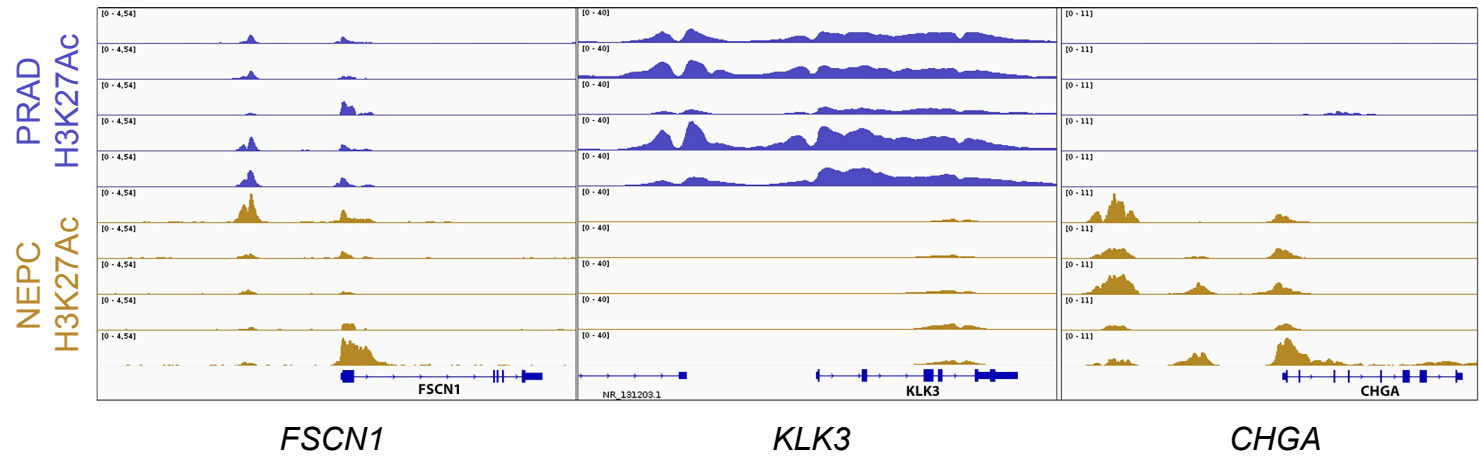

Supplement: Supplementary file 10 — Supplementary Figure S9 [file 41416_2023_2449_MOESM10_ESM.pdf]
