# Supplementary material for: Fascin-1 expression is associated with neuroendocrine prostate cancer and directly suppressed by androgen receptor
Source: Br J Cancer. 2023 Oct 24;129(12):1903–14. doi: 10.1038/s41416-023-02449-x (PMC10703930; doi:10.1038/s41416-023-02449-x)

Supplementary Figure S2

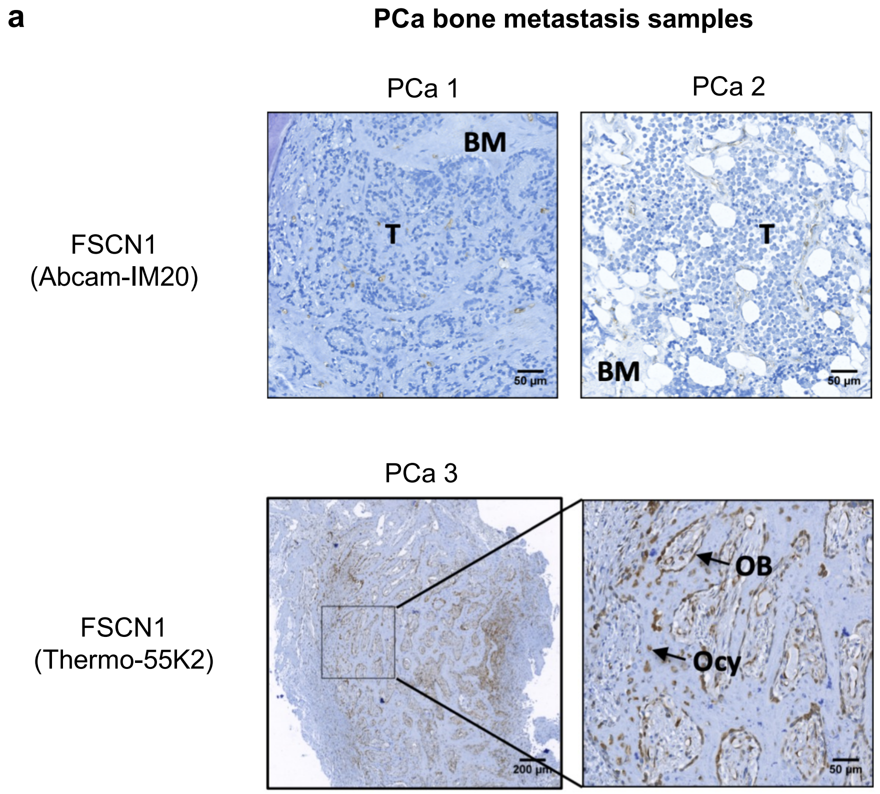

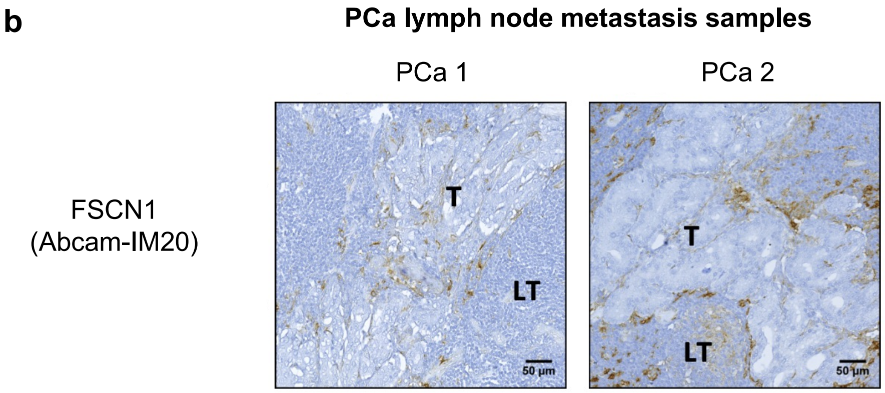

Supplement: Supplementary file 3 — Supplementary Figure S2 [file 41416_2023_2449_MOESM3_ESM.pdf]
