# Supplementary material for: Fascin-1 expression is associated with neuroendocrine prostate cancer and directly suppressed by androgen receptor
Source: Br J Cancer. 2023 Oct 24;129(12):1903–14. doi: 10.1038/s41416-023-02449-x (PMC10703930; doi:10.1038/s41416-023-02449-x)
Supplement: Supplementary file 11 — Supplementary Table S1 [file 41416_2023_2449_MOESM11_ESM.pdf]

Quantitative PCR Primers		
**Gene Name**		Sequence (5'-3')
**CHGA**	Forward	GCGGTGGAAGAGCCATCAT
	Reverse	TCTGTGGCTTCACCACTTTTCTC
**ENO2**	Forward	AGTTGCCCCTGCCTTAC
	Reverse	GAGACAAACAGCGTTACTTAG
**SYP**	Forward	TGGGGACTACTCCTCGTCAG
	Reverse	CACATGAAGGCGAACACAGC
**SOX2**	Forward	CATGGGTTCGGTGGTCAAG
	Reverse	TGATCATGTCCCGGAGGT
**BRN2**	Forward	ACACTGACCGATCTCCACGCAGTA
	Reverse	GAGGGTGTGGGACCCTAAATATGAC
**FSCN1**	Forward	AAAAGTGTGCCTTCCGTACC
	Reverse	CCCATTCTTCTTGGAGGTCA
**AR**	Forward	GCCTTGCTCTCTAGCCTCAA
	Reverse	GTCGTCCACGTGTAAGTTGC
**KLK3**	Forward	AGTGCGAGAAGCATTCCCAAC
	Reverse	CCAGCAAGATCACGCTTTTGTT
**GAPDH**	Forward	AAGGTCGGAGTCAACGGAATT
	Reverse	CTCCTGGAAGATGGTGATGG
**L32**	Forward	CAAGGAGCTGGAAGTGCTGC
	Reverse	CAGCTCTTTCCACGATGGCT

Supplementatry Table 1
