# Supplementary material for: Fascin-1 expression is associated with neuroendocrine prostate cancer and directly suppressed by androgen receptor
Source: Br J Cancer. 2023 Oct 24;129(12):1903–14. doi: 10.1038/s41416-023-02449-x (PMC10703930; doi:10.1038/s41416-023-02449-x)
Supplement: Supplementary file 12 — Supplementary Table S2 [file 41416_2023_2449_MOESM12_ESM.pdf]

Chromatin-Immunoprecipitation-Quantitative PCR (ChIP-qPCR) Primers		
		Séquence (5'-3')
**a**	Forward	GGTGGAGGTCGTAGCATTGT
	Reverse	AGTAGAGACGGGGTTTTGCC
**b**	Forward	ATCGGTCAGAACAGTGCTCG
	Reverse	TGGATTCAGGCATGCACCA
**c**	Forward	GGCAAAACCCCGTCTCTACT
	Reverse	CAGCAAGGTGAATTGGAGCAC
**d**	Forward	CAAGATTGTACCACCGCCCC
	Reverse	CTCCTCCCTGAACCACCTCT
**e**	Forward	ACGTCACTATGAGCCACCAC
	Reverse	ATCCTGCTTTCACTGAGGGC
**Prom KLK3**	Forward	TGGGACAACTTGCAAACCTG
	Reverse	CCAGAGTAGGTCTGTTTTCAA
**Control region**	Forward	CCTGGAGGGCTTGGAGAT
	Reverse	ATCCTACGGCTGGCTGTGA

Supplementary Table 2
