# Supplementary material for: Fascin-1 expression is associated with neuroendocrine prostate cancer and directly suppressed by androgen receptor
Source: Br J Cancer. 2023 Oct 24;129(12):1903–14. doi: 10.1038/s41416-023-02449-x (PMC10703930; doi:10.1038/s41416-023-02449-x)
Supplement: Supplementary file 13 — Supplementary Table S3 [file 41416_2023_2449_MOESM13_ESM.pdf]

PCA samples	Histology	Gleason score	TNM Stage*			Metastatic status at biopsy	Pre-biopsy PSA (ng/mL)**	Abiraterone prior to biopsy	Enzalutamide prior to biopsy	LHRH agonist/LHRH antagonist prior to biopsy
			T	N	M					
NEPC-1	Small cell neuroendocrine carcinoma	NA	T4	N1	M1b	Yes (bone)	3.4	No	No	No
NEPC-2	Small cell neuroendocrine carcinoma	NA	T4	N1	M1c	Yes (liver)	0.3	No	No	Yes (hormono-radiotherapy >10 years ago)
NEPC-3	Small cell neuroendocrine carcinoma	NA	T3b	N1	M1b	Yes (bone)	33	No	No	No
NEPC-4	Poorly differentiated adenocarcinoma with neuroendocrine contingent.	9 (4+5)	T3a	N1	M1	Yes (bone)	2,6	No	No	Yes (1 month)

Supplementary Table 3: Histo-pathological Characterization of NEPC samples (n=6)
PSA: prostate specific antigen, NA : not available
*According to AJCC-TNM
**According to the patients' laboratory standards

Supplementary Table 3
